# Triangular Test of *Amanita* Mushrooms by Using Electronic Nose and Sensory Panel

**DOI:** 10.3390/foods8090414

**Published:** 2019-09-14

**Authors:** Francisco Portalo-Calero, Patricia Arroyo, José Ignacio Suárez, Jesús Lozano

**Affiliations:** 1 Escuela de Ingenierías Industriales, Universidad de Extremadura, 06006 Badajoz, Spain; pacoportalo@unex.es (F.P.-C.); parroyoz@unex.es (P.A.); jmarcelo@unex.es (J.I.S.); 2 Instituto Universitario de Investigación en Recursos Agrarios (INURA), Universidad de Extremadura, Avd. De la Investigación, 06006 Badajoz, Spain

**Keywords:** smell, sensory analysis, triangular test, mycology, *Amanita*, electronic nose

## Abstract

This work aims to advance understanding of the differentiation of mushroom species through electronic devices that use sensors of various technologies and techniques for pattern recognition, comparing mainly volatile substances that emanate from them. In this first phase, the capacity of human olfaction to differentiate between the smell released by different wild mushrooms of the genus *Amanita* was analyzed by means of a triangular sensory test, comparing later the data to those obtained for the same samples with an electronic nose in a similar test. The results, still very preliminary, encourage imagining the wide application that these techniques will have and the feedback that this application can suppose for the training of the sense of human olfaction.

## 1. Introduction

Recent studies have seemed to indicate that the fact that humans have fewer olfactory receptors than some animals [1] does not necessarily mean that they have a worse olfactory sense [2]. It is currently considered that human olfaction is capable of detecting many more of the 10,000 smells than popular wisdom suggests [3]. This contradicts, to a certain extent, its long-suffering reputation as being only the fifth sense [4], since our ancestors abandoned the condition of hunter-gatherers. In the 19th century, the French scientist Paul Broca considered the human olfactory system to be primitive and its evolution to be inversely proportional to that of intelligence [2].There is also an added cultural problem, and according to References [4,5], this indicates that “...One could speculate that we have lost the ability to describe odors with the shift to agriculture...the inability to name odors is not a biological limitation, but also a cultural one”. Possibly this limitation in defining odors through language has favored generalization when classifying them. For example, sensory properties as broad as sweet, fruity, syrupy, earthy, and antiseptic have long been used to define organoleptic characteristics in the classification of mushrooms [6]. On the other hand, it is known that certain animals develop the capacity to detect volatile substances and thus find traces of people, smell diseases [7], locate explosives [8], etc. Among the natural aromas that they are able to differentiate between are those released by some types of mushrooms, which are used traditionally in their harvest once trained for it. This circumstance suggests the possible existence of different odorous nuances between the different species of mushrooms beyond those that are currently recognized by the human sense of smell. 

The literature on fungi, mainly the field guides that include a card for each species evaluated, contains a macroscopic description of mushrooms, where the smell attribute is generally found within the meat section. However, although they sometimes provide important data for the classification of certain species, the description is usually very vague and generalist, so it cannot be considered, by itself, definitive in determining the species in question. If we focus on a review of the information provided by these guides on the wild mushrooms analyzed in this work, in all of the genus Amanita, which is located in the southwest of the Iberian Peninsula, uniformity has been detected in odor assessments. However, this is not complete, and the lexical deficiency is noted in the definition of the olfactory character of each of the species of mushrooms. For example, the Amanita caesarea, a well-known and gastronomically appreciated mushroom, is indicated in different guides as follows: “little that is appreciable” [9], “pleasant mushroom smell” [10], “smooth and pleasant odor” [11], or “almost nil” [12].

**Figure 1:**
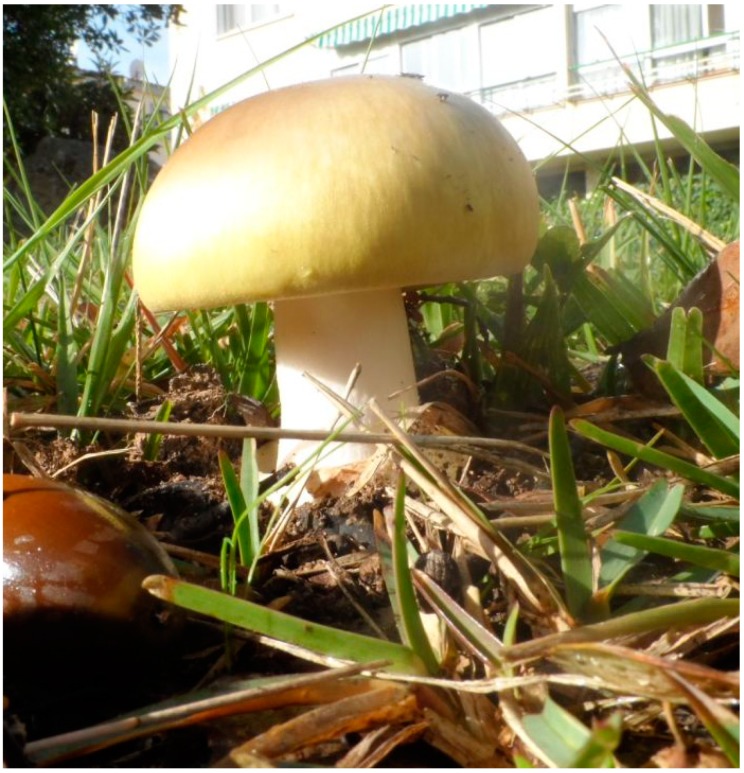
*Amanita phalloides*, cause of the deadly phalloidin syndrome.

The use of this methodology of descriptors, so general and well known, can be efficient among those species in which there is a notable odorous difference. Nevertheless, they greatly limit the ability to distinguish one species from another when the character of its odor is nearby. This need for taxonomic classification, apart from the scientific point of view, is absolutely necessary for a hypothetical consumer, as there are edible individuals and others with a high degree of toxicity. Obviously, mycologists, amateurs, and experts use other markers in their classification, but sometimes the difficulty of unequivocal identification of a fungus through its phenotypic characteristics (morphology and physiology) is extremely difficult [13]. However, for example, in recent years in Spain, approximately 0.2% of consultations in which the Toxicological Information Service issued a report [14] have been related to mushrooms. Although this did not always mean serious poisoning, in some cases (Figure 1), the consequences were dramatic [15].

At this point, two questions could be asked: Could humans really differentiate certain species of mushrooms by smell? In addition, could artificial systems be useful in this task? Sensory evaluation was used to try to answer the first question. The use of this standardized tool, which is of great importance in food research and development [16] and which has already been used in the recognition of mushroom characteristics [17], has proven to be a good choice in obtaining the indicators of human capacity to differentiate between odors. Recent studies have used triangular testing, along with other techniques, to identify key aromatic compounds as well as ketones and alcohols in some types of fungi [18]. With respect to the second question raised, we can point out that Reference [19] demonstrated that the species of fungi of the genus *Penicillium* could be discriminated between by means of an analysis of volatile secondary metabolites. Moreover, a gas chromatography–mass spectrometry (GC–MS) evaluation of different species of fungi [20,21,22] made evident that the chemical structures of many of these metabolites are similar to those of known fragrance chemicals. In Reference [6], a table showed more than 150 types of aromatic components present in mushrooms related to one or several odors.

On the other hand, the field of research on artificial sensory systems, more specifically that developed by so-called electronic noses, has demonstrated its potential to identify and solve problems in cosmetic production processes, food industries [23], chemical engineering, environmental monitoring [24], explosives detection, and medical diagnostics and bioprocesses [25]. There has also been electronic nose analysis in the literature that has differentiated between the hedonic tones of mushrooms [26], and there have been electronic noses in conjunction with GC–MS techniques to determine the degree of drying of cultivated mushrooms [27]. It is important to note that the applications developed with these devices are increasingly accessible and portable [28,29].

This work is a first step within a research project that aims to advance understanding of the differentiation of mushroom species through electronic equipment and artificial intelligence (AI) techniques, comparing mainly volatile substances that emanate from them. Later on, the aim is to develop devices that discriminate between wild and commercial mushrooms, taking into account the possible harmful substances present in these mushrooms. In this experiment, once the presence of characteristic components of the aroma of mushrooms was confirmed by GC–MS techniques, the same samples analyzed in the sensory test were subjected to an analysis using an electronic nose. Then, a comparative evaluation of the results was subsequently carried out.

## 2. Materials and Methods 

### 2.1. Samples

Before starting the collection work, the possible pathologies derived from the manipulation or inhalation of spores of toxic mushrooms were studied, as well as the possible influence that this manipulation could have on the information obtained. Once the corresponding consultations had been carried out, it was clearly established that the handling of toxic mushrooms does not, in itself, entail a risk to the health of the people who smell or touch them [30]. The use of neutral-odor latex gloves was established throughout the preparation process in order to avoid allergic reactions or possible contamination of the samples. 

The statistical population upon which this work was carried out was defined by 6 species of mushrooms of the genus *Amanita* (Table 1). These species are present in different areas of the southwest Iberian Peninsula (Table 2) and were collected during the autumn of 2018. At that moment, all selected mushrooms were classified by species and stage of ripeness (young, optimal, and mature) by at least two independent experts.

For the tests, the hats [31] from individuals of optimal mushrooms were chosen. In a period of time not greater than four hours from the harvest, they were put under a process of freezing to −24 °C. Prior to freezing, they were quartered, and each sample was weighed and stored in coded odorless bags. The major parts were allocated for triangular sensory analysis and the remaining parts for triangular analysis with an electronic nose. Some individuals were assigned to perform a GC–MS analysis of all mushroom species participating in the study.

### 2.2. GC–MS Measurements

In order to verify the existence of differential volatile compounds in each of the 6 species of mushrooms studied in this work, some analyses had previously been carried out using 0.5 g of each of them. The technique solid-phase microextraction followed by gas chromatography–mass spectrometry (SPME-GC–MS) was applied. A GC–MS instrument manufactured by BRUKER (Billerica, MA, United States) was used for this purpose. An 85-µm Carboxen/Polydimethylsiloxane (CAR/PDMS) fiber was exposed to the headspace, while the sample was shaken at 40 °C for 30 min. Subsequently, desorption took place in the injection port for 10 min. The identification of the compounds was done based on the results provided by the National Institute of Standards and Technology(NIST) spectroscopy database [32].

The temperature program used consisted of three phases. First, the oven temperature was set at 45 °C for 1 min. It was then increased to 200 °C at a rate of 5 °C/min. Finally, it increased by 20 °C /min until it reached 250 °C, a temperature which was maintained for 5 min. In addition, between the different injections, a cleaning procedure of the SPME fiber was carried out. To do this, the SPME fiber was kept at 300 °C for 30 min.

### 2.3. Triangular Sensory Test

Discriminatory sensory tests are those in which it is not intended to know the subjective sensation produced by the individual tested, but to establish the differences that may exist between two or more samples [33].

In this study, a particular triangular sensory test was carried out. In this test, in addition to setting a level of significance to validate the statistical differentiation between the odors of the samples, it was also intended to obtain more specific probabilistic data for each number of correct answers. In this way, the results could be compared to those obtained in the tests with the electronic nose.

Four sessions were held over a month, with a total of 16 combinations or triangles (4 per session), in which all the participating species were compared to each other (Table 3). In each triangle, three samples were presented simultaneously. They were chosen from among the six species under study, of which two belonged to the same mushroom and the remaining one to a different one. In all the tests, 25 (not trained) consumer judges participated forming a very heterogeneous group in mycological knowledge (experts, amateurs, students, curious people …), gender and age. All of them attended the Jornadas Micológicas 2018, held during the month of November at Centro de Profesores y Recursos in Badajoz (Spain).

**Figure 2:**
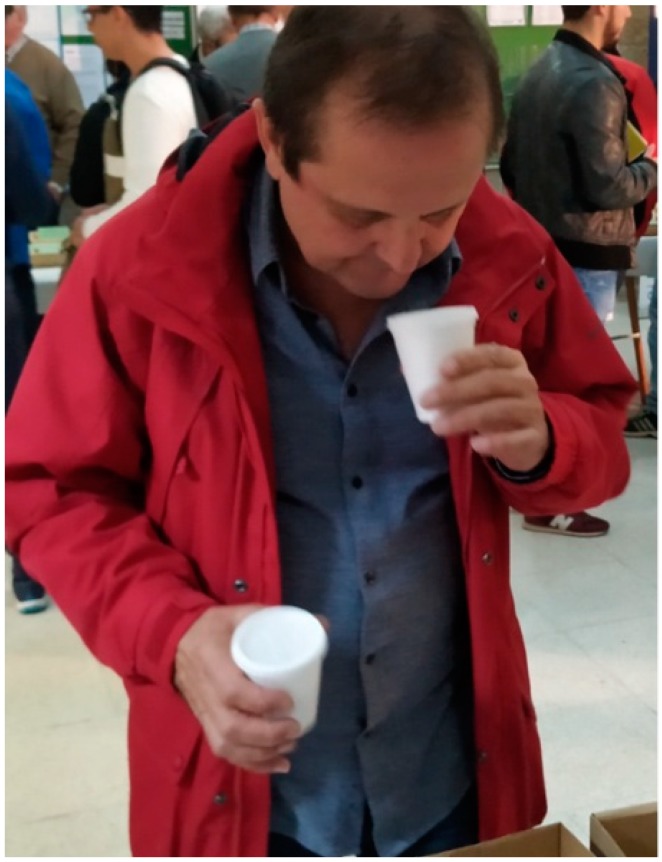
Judge from the sensory panel participating in the triangular sensory test.

After smelling the mushrooms, the judges were asked to mark, on the test sheet provided, the number corresponding to the one they considered different from each of the triangles of the session (Figure 2). In order to avoid possible stimulus errors, equal odorless containers were used, which made it impossible to recognize the mushrooms by any other sense than smell. The answers were coded with four-digit random numbers to avoid errors of expectation [16].

### 2.4. Electronic Nose

**Figure 3:**
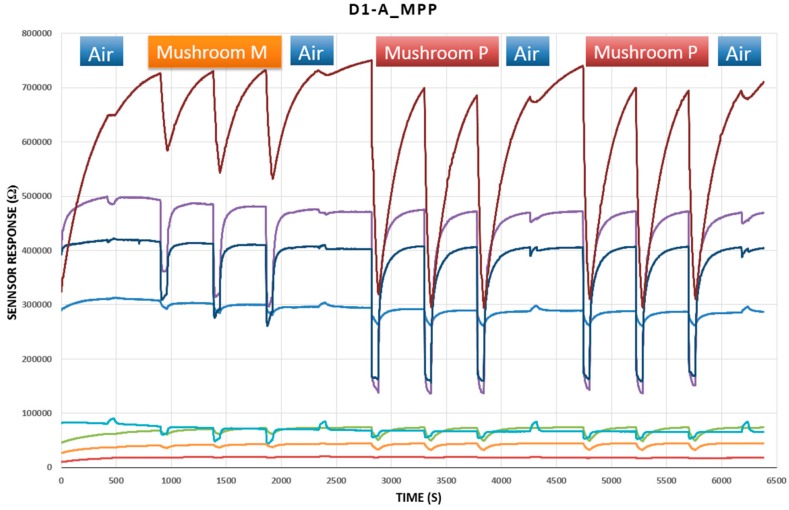
Curve of the response of the sensors to the D1-A_MPP triangle test.

A home-developed low-cost electronic nose was used for measurements. The Wireless Electronic Nose (WINOSE 6) (Figure 3) was designed and developed jointly by the Spanish Council for Scientific Research (CSIC, Madrid, Spain) and the University of Extremadura.

**Figure 4:**
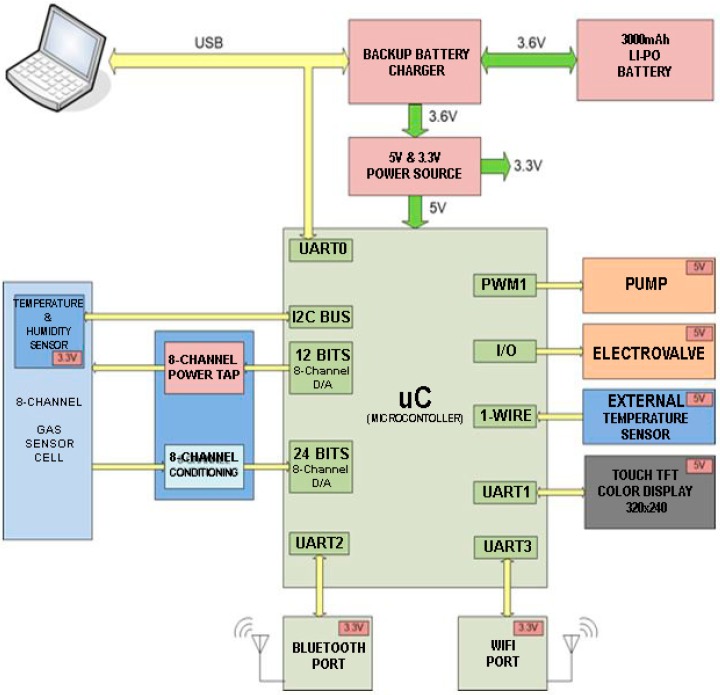
Block diagram of the electronic nose.

The electronic nose was divided into the following functional parts: an aroma extraction system, a detection system or sensor, a control and instrumentation system, and a data and signal processing system. A block diagram of the WINOSE 6 is shown in Figure 4.

It had an array of 8 interchangeable sensors that were controlled by a dsPIC33FJ128GP306 microcontroller from Microchip. In addition, the electronic nose contained integrated controls and instrumentation electronics, rechargeable batteries, a touch screen, and an IEEE 802.11 transceiver for wireless communications. The e-nose was equipped with an Liquid Cristal Display (LCD) touch screen where the responses of the sensors and the main measurement parameters were shown.

The software that controlled the entire process was developed with the NI LabVIEW program from National Instruments. It made it possible to preset all of the parameters of the analysis (e.g., the sensor heater temperatures, pump power, or absorption and desorption times).

The metal oxide (MOX) gas sensors implemented in the system, which have already been successfully used in the recognition of fungi, are shown in Table 4, which indicates the manufacturers and the preset temperature for the heaters. The MiCS4514 was composed of two sensors that were included in the same package, while the MiCS6814 was composed of three. The rest included only one sensor per chip. The sensors were located inside a cell connected to a three-way solenoid valve (SMC S70_ES), which permitted selection between two inputs: one for the sample to be measured and the other one for a reference gas (in our case free air), which was passed through an active carbon filter to obtain the reference baseline. The electronic nose also had relative humidity and temperature sensors in the same package (Sensirion SHT21, Sensirion, Stäfa, Switzerland) and a micropump (RietschleThomas model 2002, Gardner Denver, Milwaukee, WI, USA).

#### 2.4.1. Sampling Method

**Figure 5:**
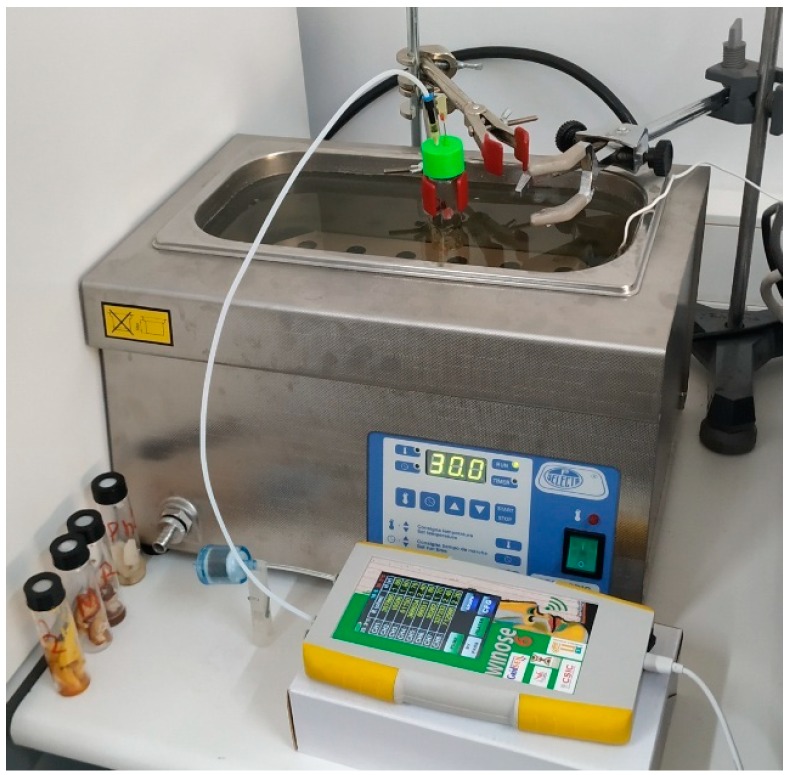
Test of mushrooms with the electronic nose WINOSE 6.

Static headspace with a transfer of effluent technique was used for volatile extraction [34]. Each piece of sample to be analyzed, after being thawed, was placed in a 20-mL glass vial and sealed with a septum and screw cap to allow the injectors to enter. The vials were immersed in a thermostatic bath at 30 °C (Figure 5). In order to reset the same position (the inclination and penetration of the injection needles) in all analyses, a cap with stop guides was designed and used.

The analysis began with progressive heating of the sensor surfaces, and once they reached their stabilized reference temperatures (Table 4), the programmed tasks began. Each data acquisition cycle consisted of two parts. First was a 7-min desorption period, during which filtered free air was injected into the cell where the sensors were located. Every second, the system took a reading of the resistive value supplied by each sensor, thus obtaining the reference baselines. After that period of time, the valve that allowed the absorption of volatiles of the sample to be analyzed was activated for 1 min, taking one reading per second. In order to make the experiment as similar as possible to the triangular sensory test, 3 absorptions were made for each mushroom sample, and a filtered free air absorption was always made before, between, and after each sample (Figure 3).

#### 2.4.2. Preprocessing

To select the descriptive parameters of the resistive values obtained by the sensors, the fractional baseline manipulation procedure was used. It consisted of dividing the difference between the resistive value obtained at the reference baseline and the response of each sensor to the volatiles emanating from the corresponding mushroom sample by the reference baseline (Equation (1)). In this way, the multiplicative drift was eliminated, generating a dimensionless and normalized response [35]:

(1) $$Ri=\frac{R_{ref}-R_{aroma}}{R_{ref}}$$

### 2.5. Statistical Analysis

The objective of this work was to compare the discriminative capacity (between the analyzed samples) that the panelists and the electronic nose had. To this end, analogous guidelines were maintained in the two procedures, both in terms of data retrieval, as commented on previously, and in terms of statistical analysis.

According to these criteria and given that the participating judges did not have previous training, it was considered that the device results should be obtained through the use of an unsupervised learning method. In addition, this was all the more convenient as there were very few readings for each triangle, which greatly limited the training process. In order to validate the statistical accuracy in the discrimination of the analyzed mushrooms, a level of significance was established in both cases, α = 5%, which is the level usually used in this type of test [36]. As this was a comparative study, the level of statistical confidence was also assessed (1–α), which was derived from the number of correct answers of each triangle analyzed by means of the calculation of the probabilistic value *p*-value.

#### 2.5.1. Sensory Analysis

The null hypothesis in a triangular test establishes that the probability of randomly choosing a different sample is *p* = 1/3 [37]. A null hypothesis (H_0_) was established: if we odorously compared two different mushrooms, A and B, of the genus *Amanita* of the species *A. caesarea*, *A. phalloides*, *A. rubescens*, *A. pantherina*, *A. muscaria*, or *A. citrina*, which were collected from different points of the southwest of the Iberian Peninsula during the month of November 2018, we would not find odorous differences between them (Equation (2)). If H_0_ were rejected, we would not reject the alternative hypothesis H_1_ (Equation (3)), which would give credibility to the existence of odorous differences between the individuals analyzed:

(2) $$H_{0}:\mu_{A}=\mu_{B}$$

(3) $$H_{1}:\mu_{A}\neq \mu_{B}$$

In a first approximation, the tables published by Reference [38], which were based on a binomial distribution, were used. There, the minimum number of correct answers was indicated, depending on the number of participating judges, for establishing significant differences between the two samples with the prefixed level of significance α. Second, in order to compare this to the results obtained by the electronic nose, the *p*-value of each triangle was calculated as a function of the number of correct answers. Equation (4) was used, together with Equation (5), where the probabilities of a higher number of correct answers than obtained were assessed. Table 5 indicates the *p*-value obtained for a certain number of correct answers by 25 judges, indicating the rejection of H_0_ with α = 0.5 and also the limits for α = 5%, α = 1%, and α = 0.1%:

(4) $$P\left(y\right)=\left(\begin{array}{c} n\\ y \end{array}\right)p^{y}{\left(1-p\right)}^{n-y}$$

(5) p-value=P(y)+P(y+1)+⋯+P(n)

where *n* = 25 and *p* = 1/3 at *y* = no. of correct answers.

#### 2.5.2. Electronic Nose Data

**Figure 6:**
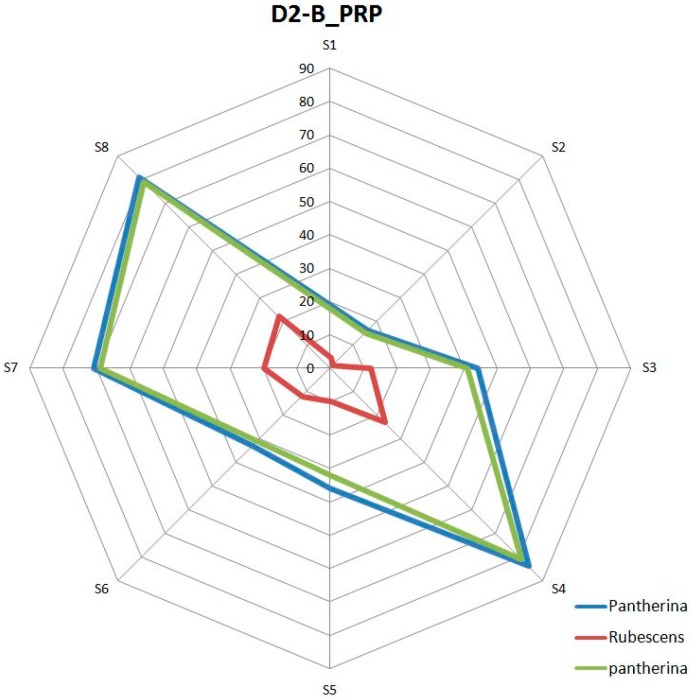
Graphical representation of the aromas obtained from the triangle D2-B_PRP.

**Figure 7:**
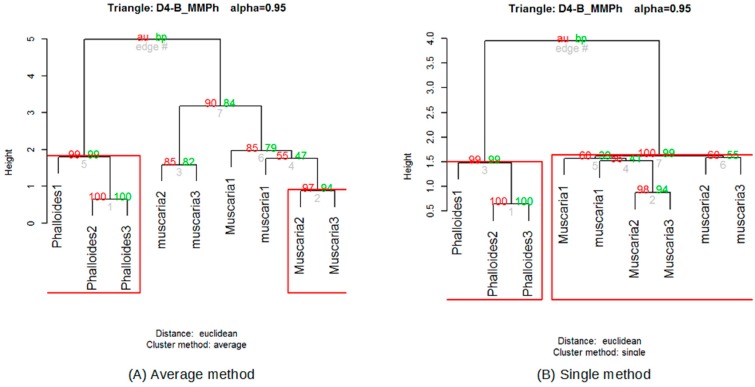
Differences in dendrograms for the same data with average (**A**) and single (**B**) grouping methods.

Once the data supplied by the sensors were preprocessed, the fingerprints of the volatile organic compounds (VOCs) [39] present in each mushroom were obtained. As defined by Reference [40], the term volatile organic compounds (VOCs) refers to those organic compounds that are present in the atmosphere as gases but that would be liquids or solids under normal conditions of temperature and pressure. In this same publication [40], the enhancement of the sensing performances of MOX gas sensors for detecting toxic gases and VOCs is also highlighted. As has been mentioned, mushrooms generate a great diversity of these VOCs [6]. With these data, after averaging, the aroma obtained from each triangle was represented by means of radial graphs (see Figure 6). The automatic classification of these fingerprints was performed using the multivariate statistical agglomerative method hierarchical cluster analysis (HCA) [41]. The HCA refers to an unsupervised learning technique that aims to find patterns or clusters within a set of observations. The partitions are established in such a way that the observations that are within the same group are similar between them and different from those of other groups. For the hierarchical cluster analysis, the *hcluster* function (https://www.rdocumentation.org/packages/amap/versions/0.8-16/topics/hcluster), which belongs to the statistical analysis program R (https://www.r-project.org/) was used. Before the analysis, a series of tests was carried out to determine the most suitable methods for the analysis of the data obtained, as this is not a trivial matter and should be checked when an HCA is applied [41]. From these checks, the Euclidean method was chosen for distances and the single method [42] for clusters (Figure 7).

In order to obtain the *p*-value of each grouping, the R *pvcluster* library was used [43] with a significance level of α = 0.5. 

Once the dendrogram was created, it had to be validated, assessing to what extent its structure reflected the original distances between observations. One of the most commonly used criteria for that is the cophenetic correlation coefficient of the dendrogram [44]. If a value close to the unit is obtained, this means that there is a good hierarchical structure between the elements analyzed. Some publications have indicated that a good partition must have a cophenetic correlation of at least 0.85 [45].

## 3. Results and Discussion

### 3.1. GC–MS

The values shown in Table 6 for each compound are absolute values of measured areas, so they do not correspond to a certain concentration. Some compounds are not detected (ND).

Nowadays, it is generally accepted that compounds containing eight carbons are the primary volatile contributing to the taste of fungi. Compounds of this class include 3-octanol, 3-octanone, 1-octen-3-ol, etc. [46]. It can be observed that they appeared in different proportions in the chromatographic analysis carried out, which indicated a possible odorous variation in each of the species analyzed.

### 3.2. Sensory Analysis

Table 7 shows the results of the triangular sensory analysis for a significance level of α = 0.5 expressed as a function of the confidence level (1–α). The *p*-value corresponding to each triangle is enclosed in parentheses, depending on the number of correct answers.

From these results, it can be concluded that in over 87% of cases, H_0_ could be rejected and H_1_ accepted. When the null hypothesis H_0_ is rejected, there is statistical evidence that the alternative hypothesis H_1_ is acceptable. On the other hand, if it is not rejected, there is no statistical evidence to accept it. Because the *p*-value of the triangles in which H_0_ was accepted was very close to α, a type II error was likely to have occurred. In other words, the null hypothesis was accepted even though it was false. In both cases, it was very possible that the sensory test did not have sufficient contrast intensity to determine the stipulated percentage of odor difference. This could be solved by increasing the number of judges [47]. 

To sum up, it could be statistically affirmed that we humans are capable of differentiating between mushrooms through smell, and therefore, we can begin to answer affirmatively the first of the questions presented by this research.

A further interesting aspect to observe is how sensory results improve over time. Perhaps it was due to the increased motivation shown by the participating judges during the development of the trials and because of the eventual learning from the tasting practice.

### 3.3. Electronic Nose Results

#### 3.3.1. Graphical Results

**Figure 8:**
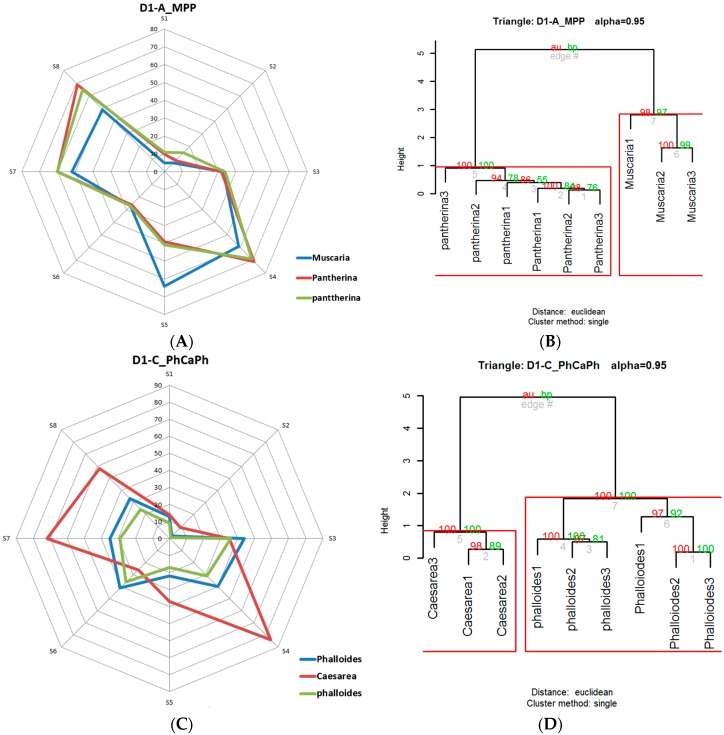

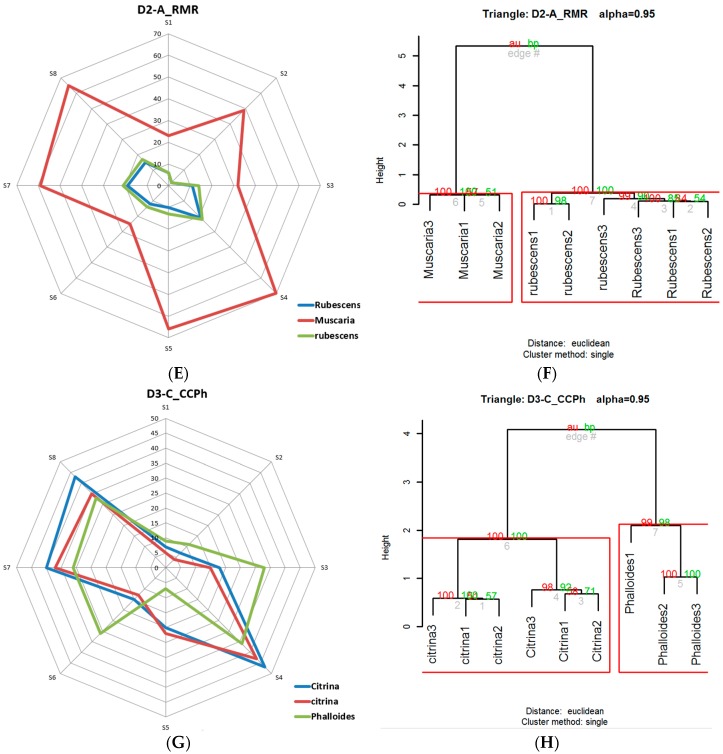
Odor fingerprints and dendrograms of the triangles: D1-A_MPP (**A**) (**B**), D1-C_PhCaPh (**C**, **D**), D2-A_RMR (**E**) (**F**), and D3-C_CCPh (**G**) (**H**).

The response curve of each sensor, which was obtained directly from the e-nose, anticipated the possible volatile differences between the two types of mushrooms analyzed in each triangle. These differences were more accurately visualized once preprocessed and averaged, as shown in Figure 8, where the radial graph is shown next to the dendrogram of the triangles D1-A_MPP, D1-C_PhCaPh, D2-A_RMR, and D3-C_CCPh. The sensors used, despite having a long recovery time (desorption period), showed a fairly stable performance. It should be noted that the e-nose tests were performed under very similar humidity and temperature conditions. Cyclically, calibrations were carried out with 10% ethanol, in which good temporal repeatability in the responses was observed. It was also observed that some sensors had a wider response margin than others. Some of the previous considerations are also reflected in Figure 8, where, for instance, we can observe that the sample of *Amanita muscaria* from the D-1_MPP test was very similar to the one obtained from the D2-A_RMR test. 

#### 3.3.2. Results Obtained through the Multivariate Statistical Method HCA

Table 8 shows the results of all analyses performed. It was determined that only two of the sensory trials (D3-A_PhPPh and D4-A_CaPCa) and one of the electronic nose trials (D4-A_CaPCa) did not reject the null hypothesis at the established significance level.

**Figure 9:**
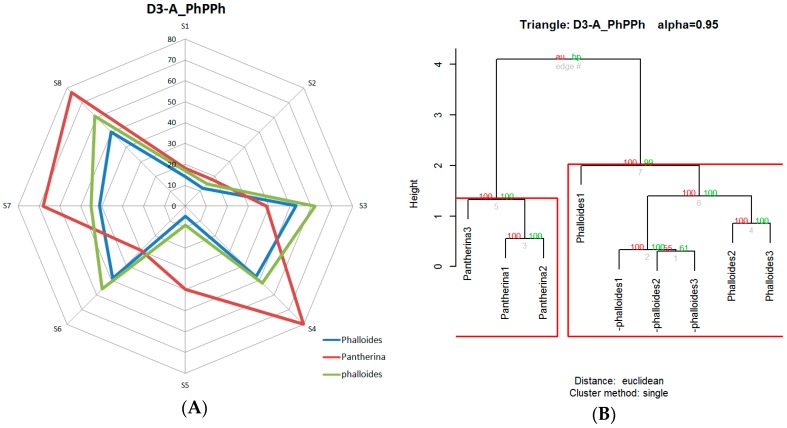
Radial graph (**A**) and grouping dendrogram (**B**) of triangle D3-A_PhPPh.

These results evidence that, in most trials, the electronic nose had a high rate of odor discrimination between samples. Over 80% of the triangles had a confidence level above 99%, indicating that, potentially, a lower significance level could have been applied if wider sampling had been performed. The radial graph (A) and the grouping dendrogram (B) of the triangle D3-A_PhPPh, which was one of the triangles exceeding 99%, are shown in Figure 9. Notice that the graphical difference is perfectly in accordance with the hierarchical grouping. The data obtained by the hierarchical clustering analysis were evaluated (Table 8) using the cophenetic correlation coefficient method.

**Figure 10:**
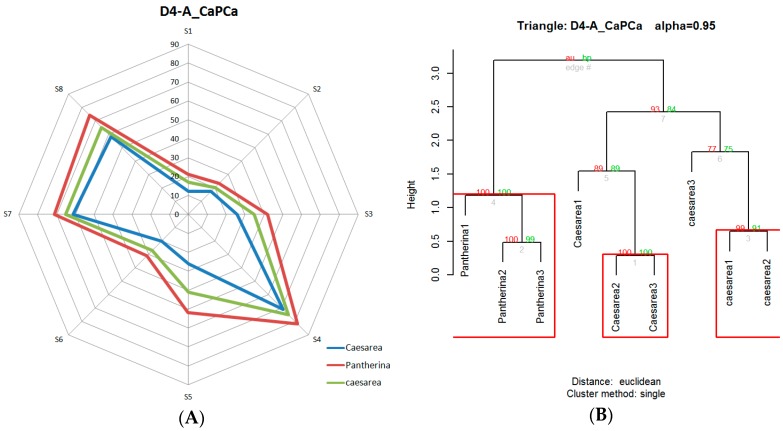
D4-A_CaPCa: the only triangle that did not reject null hypothesis in the hierarchical cluster analysis (HCA) analysis. (**A**) Radial graph; (**B**) dendrogram.

The D4-A_CaPCa triangle was the only one that did not reject the null hypothesis, with an associated *p*-value of 0.1147. In the radial graph of Figure 10A, the discrimination between both types of mushrooms was not clear. This was confirmed by the detection of more than two groups by the *hcluster* function, which evidenced the impossibility of differentiating one mushroom from another by grouping, as observed in Figure 10B. In Table 6, where the aroma compounds of mushrooms are indicated, it can be seen that *A. caesarea* (Ca) and *A. pantherina* (P) had the closest profile. This may suggest that there are similarities between the volatile organic compounds that emerge from them. However, in contrast, in the corresponding sensory trial, a high level of discrimination was obtained.

Despite this particular situation, it could be statistically affirmed that the electronic nose was able to differentiate between the vast majority of the analyzed mushrooms with enough clarity. This answers affirmatively the second question presented: machines can help us to find odorous differences in mushrooms.

### 3.4. Results Comparison

**Figure 11:**
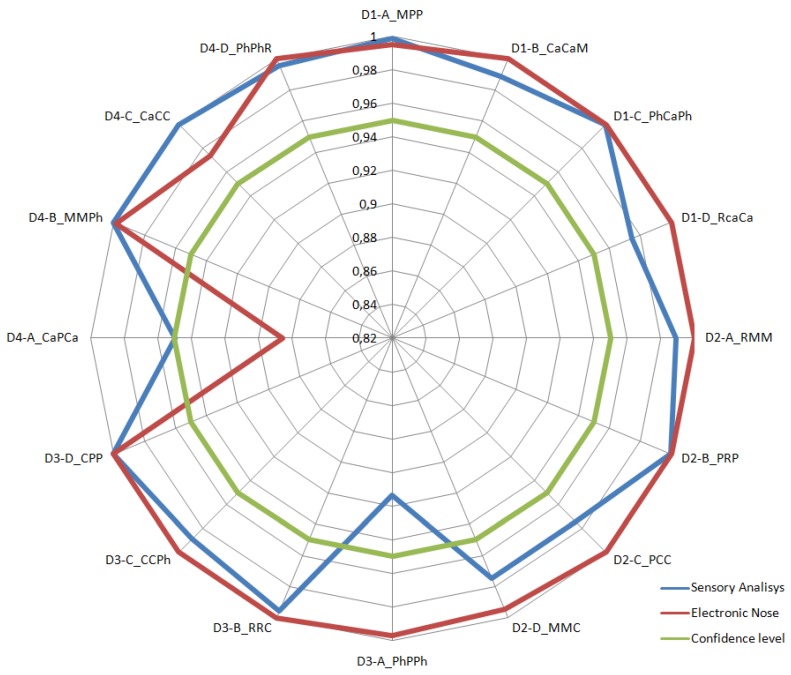
Comparison of the results of the triangular sensory test to those obtained by the electronic nose.

In the “% Difference” column of Table 8, the *p*-value corresponding to the electronic nose was subtracted from the *p*-value corresponding to the sensory test. The six positive results indicate an equal or better performance of human olfaction in olfactory differentiation of these triangles, while the negative results indicate the opposite. It can be observed that human smelling discriminated with a confidence level of over 99% in 8 triangles, while the machine achieved 99.9% in 10 triangles. These odorous discrepancies between humans and machines, which are shown in Figure 11, suggest that they move in a slightly different odorous spectrum. Figure 11 also reveals an improvement in the results of sensory analysis over time, as previously outlined.

## 4. Conclusions

From a statistical point of view, the capacity of both human and electronic devices to discriminate between the species of wild mushrooms analyzed in this study was demonstrated. Now, although the study fulfilled the proposed objective, it was limited to a small number of species. In addition, they were of a single genus, were localized temporally and geographically, and were discriminated against (but not recognized). This should be the next working approach.

The learning detected in the discrimination of mushrooms through the sense of human smell at the end of the sensory analysis supports studies that suggest its potential, although this has already been demonstrated in other fields, such as oenology, where an interesting wheel of aromas has been developed [48]. There is a need to work on the development of a similar tool in the field of mycology, which would provide olfactory education aimed at (together with other markers) the recognition of species of mushrooms, especially toxic ones. A limitation in this aspect is the lack of specialized tasters in mushrooms, which are needed to progress in sensory evaluations with a more selective approach.

With regard to the development of electronic noses, support for the continuous improvement of different technologies of gas sensors and continuous research on possible applications could allow for the production of devices that recognize mushrooms by smell, for example, the edible *Amanita ponderosa*, which is very characteristic for its earthy smell and is sometimes confused with the *Amanita verna*, which is odorless and very toxic. The risks involved in this type of classification mean that the methods used must be totally contrasted with and complemented by other mushroom recognition tools. A further future application could be to characterize the state of ripeness of edible mushrooms.

Other issues in which advances are required are the collection of sensor data and their specificity for the analysis of volatile compounds present in mushrooms, where there is a lack of information in the corresponding datasheets. The preprocessing technique applied in this study (fractional baseline manipulation) does not consider several features of the signal obtained directly from the sensor, and they may provide relevant data for the discrimination of all types of volatiles. 

To conclude, it should be noted that, as emerging research and new applications have revealed, electronic noses, from which valuable information will be obtained in a wide range of environments, will not take long to become an integrated element in our everyday devices. In addition, it is highly desirable that they could be used to promote the development of the smell sense of humans by supplying a feedback line to improve responses to different odorous sensations. As a result, it would be possible to increase olfactory capacity and therefore human knowledge.

## Figures and Tables

**Table 1:** Edibility/toxicity and aroma of the *Amanita* species analyzed.

Mushroom	*A. rubescens* (R) ^1^	*A. pantherina* (P)	*A. citrina* (C)	*A. muscaria* (M)	*A. phalloides* (Ph)	*A. caesarea* (Ca)
**Image**	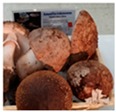	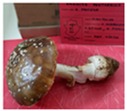	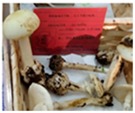	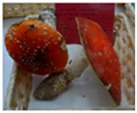	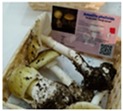	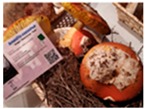
**Edibility/toxicity**	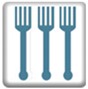	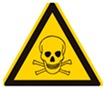	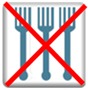	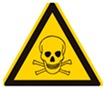	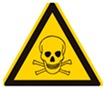	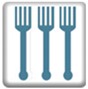
**Smell**	Odorless	Soft	Unpleasant	Unpleasant	Odorless (young)	Nice

Note: *Amanita rubescens* requires previous cooking.

**Table 2:** Most significant vegetation type and collection areas.

Collection Area	Vegetation	City
Serra de S. Mamede	Cork oak, holm oak, scrub	Portalegre (Portugal)
Comarca de La Vera	Oak and chestnut, scrub	Cáceres (Spain)
Sierra de Aracena	Cork oak, holm oak, scrub	Huelva (Spain)
Comarca de Badajoz	Holm oak, eucalyptus, scrub	Badajoz (Spain)
Monte dos Arneiros	Cork oak, scrub	Montemor-o-Novo (Portugal)
Sierra Suroeste	Chestnut, holm oak, cork oak, scrub	Badajoz (Spain)

**Table 3:** Sensorial triangle codes for each session.

Session	Triangles			
	A	B	C	D
Day 1	D1-A_MPP	D1-B_CaCaM	D1_C_PhCaPh	D1-D_RCaCa
Day 2	D2-A_RMR	D2-B_PRP	D2-C_PCC	D2-D_MMC
Day 3	D3-A_PhPPh	D3-B_RRC	D3-C_CCPh	D3-D_CPP
Day 4	D4-A_CaPCa	D4-B_MMPh	D4-C_CaCC	D4-D_PhPhR

R: A. rubescens; P: A. pantherina; C: A. citrina; M: A. muscaria; Ph: A. phalloides; Ca: A. caesarea.

**Table 4:** Sensors used by WINOSE 6.

Sensor	Manufacturer	Model	*V* heaters (*V*)		
			Minimum	Maximum	*V* Configured
S1	Cambridge	CCS801	1.2	1.8	1.8
S2	Cambridge	CCS803	1.2	1.8	1.8
S3	SGX	MICS4514 OX	1.3	1.9	1.7
S4	SGX	MICS4514 RED	2.2	2.5	2.4
S5	Figaro	TGS8100	1.8	1.8	1.8
S6	SGX	MICS6814 OX	1.3	1.9	1.7
S7	SGX	MICS6814 RED	2.2	2.6	2.4
S8	SGX	MICS6814 NH3	2	2.4	2.2

Note: Sensors S3 and S4 and S6, S7, and S8 were in the same encapsulation.

**Table 5:** Calculation of the *p*-value for a hypothesis contrast using the binomial distribution and comparison to the chosen level of significance (α = 0.5).

*n*	*y*	*p*-Value	Reject H_0_	α (%)
25	10	0.1264	NO	>5%
25	11	0.0862	NO	>5%
25	12	0.0503	NO	>5%
25	13	0.0251	YES	<5%
25	14	0.0108	YES	<5%
25	15	0.0040	YES	<1%
25	16	0.0012	YES	<1%
25	17	0.0003	YES	<0.1%
25	18	0.0001	YES	<0.1%
25	19	0.0000	YES	<0.1%

*n* = number of panelists; *y* = right answers; H_0_ = null hypothesis; α = level of significance.

**Table 6:** Volatile compounds identified by gas chromatography–mass spectrometry (GC–MS) in the mushroom species analyzed.

Compound	Samples					
	*A. rubescens*	*A. pantherina*	*A. citrina*	*A. muscaria*	*A. phalloides*	*A. caesarea*
3-Methyl-butanal	27	ND	ND	13	402	ND
2-Methyl-butanal	87.4	ND	0	ND	1300	ND
Acetic acid	ND	ND	ND	ND	ND	ND
Acetoin	ND	ND	ND	ND	ND	ND
3-Methyl-1-butanol	ND	ND	10.9	15.8	507	11.7
1-Octanol	ND	ND	ND	ND	ND	ND
Hexanal	20.5	940	6.42	28	26.3	13.9
Styrene	18.6	17.9	38.8	70.8	55.1	7.56
Heptanal	ND	4.48	ND	ND	ND	ND
Benzaldehyde	ND	ND	ND	ND	24.6	ND
1-Hept-3-one	ND	6.68	ND	84.5	ND	ND
1-Octen-3-ol	18.4	308	10.1	78.7	3.44	363
3-Octanone	159	209	280	917	153	392
3-Octanol	12.3	28.4	ND	ND	ND	19.7
Octanal	ND	11.4	ND	9.44	ND	ND
2-Ethyl-1-hexanol	40	105	ND	29.4	ND	10.91
2-Octenal	ND	2.19	ND	ND	ND	ND
Borneol	ND	ND	ND	ND	ND	ND

**Table 7:** Results of the triangular sensory analysis for a significance level of α = 0.5 and *p*-value for each triangle.

**Confidence Level**	≤95%	>95%	>99%	>99.9%
**Null Hypothesis**	Accepted	Rejected	Rejected	Rejected
**Number of Trials**	2 (12.5%)	6 (37.5%)	3 (18.75%)	5 (31.25%)
	D3-A_PhPPh	D1-B_CaCaM	D1-A_MPP	D1-C_PhCaPh
	(91.38%)	(98.92%)	(99.88%)	(99.97%)
	D4-A_CaPCa	D1-_RcaCa	D3-B_RRC	D2-B_PRP
	(94.97%)	(97.49%)	(99.60%)	(100%)
		D2-A_RMR	D4-A_CaPCa	D3-D_CPP
		(98.92%)	(99.88%)	(100%)
**Triangles**		D2-C_PCC	D4-D_PhPhR	D4-B_MMPh
		(97.49%)	(99.60%)	(100%)
		D2-D_MMC		D4-C_CaCC
		(97.49%)		(100%)
		D3-C_CCPh		
		(98.92%)		

**Table 8:** Comparative results of sensory analysis and electronic nose analysis. Negative values in the “% Difference” column indicate a better response from the electronic nose. Cophenetic correlation coefficient values.

Humans versus Machines: Triangular Analysis				
Triangle	Sensory Analysis	Electronic Nose	Cophenetic Coeff.	% Difference
D1-A_MPP	0.9988	0.9949	0.9979	0.39%
D1-B_CaCaM	0.9892	1.0000	0.8200	−1.08%
D1-C_PhCaPh	0.9997	1.0000	0.9789	−0.03%
D1-D_RcaCa	0.9749	1.0000	0.9508	−2.51%
D2-A_RMM	0.9892	1.0000	0.9244	−1.08%
D2-B_PRP	1.0000	1.0000	0.7322	0.00%
D2-C_PCC	0.9749	1.0000	0.8064	−2.51%
D2-D_MMC	0.9749	0.9948	0.7585	−1.99%
D3-A_PhPPh	0.9138	0.9970	0.9640	−8.32%
D3-B_RRC	0.9960	1.0000	0.8782	−0.40%
D3-C_CCPh	0.9892	1.0000	0.9271	−1.08%
D3-D_CPP	1.0000	1.0000	0.8064	0.00%
D4-A_CaPCa	0.9497	0.8853	0.9629	6.44%
D4-B_MMPh	1.0000	0.9981	0.9622	0.19%
D4-C_CaCC	1.0000	0.9738	0.9992	2.62%
D4-D_PhPhR	0.9960	1.0000	0.8913	−0.40%
